# Measuring the value of a digital supplemental resource

**DOI:** 10.1152/advan.00080.2021

**Published:** 2021-12-01

**Authors:** Yvonne M. Baptiste, Samuel Abramovich, Cherylea J. Browne

**Affiliations:** ^1^Department of Learning and Instruction, State University of New York at Buffalo, Buffalo, New York; ^2^Division of Science, Health, and Mathematics, Niagara County Community College, Sanborn, New York; ^3^Department of Information Science, State University of New York at Buffalo, Buffalo, New York; ^4^School of Science, Western Sydney University, Campbelltown, New South Wales, Australia

**Keywords:** learning analytics, physiology, science education, support of research, survey development

## Abstract

Supplemental resources in science education are made available to students based on the belief that they will improve course-based student learning. This belief is ubiquitous, with supplemental resources being a traditional component of physiology education. In addition, the recent large-scale transition to remote learning caused by the Covid-19 pandemic suggests an increased relevance and necessity of digital versions of supplemental resources. However, the use of a supplemental resource is entirely dependent on whether students view it as beneficial. If students in a specific course do not perceive a supplemental resource as useful, there is little reason to believe the resources will be used and are worthy of investment. Consequently, measurement of student perception regarding the effectiveness of any digital learning tool is essential for educators and institutions in order to prioritize resources and make meaningful recommendations to students. In this study, a survey was used to determine student perceptions of a digital, supplemental resource. Quantitative methods, including exploratory factor analysis, were performed on data collected from the survey to examine the dimensionality and functionality of this survey. The findings from this study were used to devise an improved, standardized (i.e., reliable and valid) survey that can be used and adapted by physi3ology researchers and educators to determine student perception of a digital supplemental resource. The survey, with known construct validity and internal reliability, can provide useful information for administrators, instructors, and designers of digital supplemental resources.

## INTRODUCTION

Physiology courses can pose challenges to students for many reasons including the extensive amount of detailed information that is expected to be learned (1–3). Using both lecture and laboratory modalities, some of the learning objectives in physiology necessitate a three-dimensional spatial understanding of anatomic structure integrated with abstract concepts, such as processes that are not visible (e.g., electrical changes in the heart and neurons, movement of substances across a plasma membrane) (4, 5). Physiology courses also use a lot of terminology based in Greek and Latin, which can be an obstacle for students who are unfamiliar with those languages (6). Consequently, conventional wisdom among physiology educators is that many students need access to supplemental resources to support their learning (7–9).

Technological advances over the past few decades have added to the number and types of digital supplemental teaching tools available in physiology education (10). Digital simulations, augmented and virtual reality, and animation are now common among the supplemental resources designed to augment a physiology student’s work in science, allied health, and related medical disciplines (11, 12). However, although these technologies have fueled an increased availability of supplemental resources, there is relatively little research that examines if these newer, digital supplemental resources are effective learning tools (13, 14). This is a particularly relevant issue since the Covid-19 pandemic has caused a major shift in pedagogy to remote learning that relies on digital resources (15, 16).

Broadly defined, supplemental resources are ungraded efforts performed outside of class time, intended to improve the students overall course performance. For example, illustrations, physical models, and experimentation are traditional supplemental tools used in human anatomy and physiology education (HAPE), which itself is a common lecture-laboratory corequisite discipline. Examples of digital, supplemental resources include simulations and digital cadavers (17). Student objectives for the use of supplementation are multifactorial and include assisting learning (8) and increasing academic performance (7). Surprisingly, even though supplementation within medical and allied health education is an essential part of the epistemology for this subject (18, 19), there is no standardized way to evaluate various supplemental learning tools. There are prior studies that use grades to measure the effectiveness of a supplemental resource (20, 21), but many academic and nonacademic factors affect grades, complicating any attribution of a grade to use of a supplemental resource.

To address the dearth of methods to evaluate digital supplemental resources, we adapted and validated a survey that measures student perception of a specific digital supplemental resource instead of relying on complex attributions to grades. There is a critical amount of research that establishes surveys as a reliable measure in science education, including the Study Process Questionnaire (SPQ), Approaches to Studying Inventory for Students (ASSIST), and the Anatomy Learning Experiences Questionnaire (ALEQ) (22–24). These research-based, standardized survey instruments focus on the broad topic of student approaches to learning and are not specific to supplementation. Because of that, the value of those surveys for informing decisions about the effectiveness of specific digital supplemental resources is limited. Research studies that examine the specific role of a single form of supplementation have utilized locally developed surveys (18, 25, 26) but with little attention to how those surveys could operate outside of their original context. The data collected from those efforts are incomparable to other efforts, meaning that we cannot aggregate the data and reach any type of general conclusions or consensus regarding digital supplemental resources. The survey we developed was designed to be adapted and used by instructors and researchers to compare student perceived value of various supplemental digital resources, allowing for both normative and relative comparisons. The survey is also designed to collect data about how a digital supplemental resource was used. We chose student perception as a proxy for the value of the resource since it can provide practical information such as perceived ease of use and perceived usefulness, which both correlate with student use (27). This broader view of assisting learning is key for understanding the relevance of supplementation since these are optional learning tools. We believe our study has wide application to physiology courses, anatomy courses, and other subjects that use lecture-laboratory corequisites.

## METHODS

### Theoretic Framework

A review of the published research on supplemental resources, conducted by the authors, suggests that the most common methods used to evaluate a supplemental resource are test grades and surveys of student perception. Test grades are sometimes favored for evaluation of supplemental resources because they suggest causation of student learning outcomes (i.e., the use of supplemental resources resulted in an increase in grade averages). However, since there are many factors that actually influence test grades (28), we argue that only research instruments that attempt to account for all of the covariates that impact grades can make a strong, causation argument related to supplemental resources. For most in situ studies or evaluations, differences in grades may or may not be the direct result of the value or lack thereof for a particular supplemental resource.

Fortunately, a standardized assessment of supplemental resources in physiology education may be possible by using a survey to measure student perception of a particular supplemental resource. A survey is a common instrument for measuring latent constructs (29), which cannot otherwise be directly quantified. In turn, the quantification of latent constructs, (e.g., beliefs, attitudes, and opinions) is then often used in large-scale data analysis (30). Although evaluating supplementary resources through student perception does not prove direct causation, prior research indicates that surveys of student perceptions can measure the characteristics (e.g., perceived ease of use, perceived usefulness, overall experience) that predict student use (31). This is an important measure because learners who perceive that a digital supplemental resource effectively provides them support will likely use the supplementation (31), bypassing a need to measure time of use directly. In addition, time of use has limited value since different students may spend different amounts of time using a resource for various impact. For example, a supplemental resource that requires little time but has a large impact on learning is possibly even more valuable than one that requires more time. Surveys of student perception can also compare different forms of supplementation, such as the student perceived strength(s) of a resource compared to another, as well as diagnose any challenges that students may encounter for a particular type of supplementation (32). Another advantage of a survey based on student perception is that it is not dependent on large data sets to be a valid determiner of educational effectiveness, as is the case for grades. Instead, evaluation of the survey tool can be done before large-scale use, ensuring construct validity and internal reliability before its use (33).

### Survey Design

If a survey of supplemental resources is to be used as a standardized measure, it is critical to consider how the survey is designed. In other words, if the design of the survey is flawed or theoretically unsound, then no amount of statistical validation is sufficient. Our design choice for the survey was a Likert scale. Typically composed of a group of four or more statements or questions, rereferred to as items (29), by asking multiple versions of an item, the scale can then be statistically validated into a standardized measure. Once standardized, researchers can compare survey scores, such as comparing the perception of different groups of physiology students about a particular supplemental resource or comparing student perceptions about different types of supplemental resources.

### Survey Dimensionality

Before a Likert scale can be used as a standardized measurement, it must be tested to see if it is functioning as intended, if it measures the intended latent construct and not unintentionally measures multiple undefined or weakly related constructs (34). Since a Likert scale is designed to measure a single concept or idea (i.e., unidimensional), there should only be one factor statistically detected within the Likert items composing that scale.

While there are multiple statistical analyses that can explore the dimensionality of a survey, principal axis factoring was chosen as a way to perform an exploratory factor analysis (EFA) on the survey data. EFA was performed rather than confirmatory factor analysis in this case because although the scale was previously implemented and published, the dimensionality of the scale was never mathematically established. Therefore, EFA was used to determine if the structure of the six survey items was a Likert scale. Principal axis factoring was used to examine if different variables (e.g., Likert items) are driven by the same or different latent constructs, known as factors (34). EFA can reveal if there are underlying associations among items in a scale and identify the number of factors that contribute to the measure (i.e., factor extraction). If a single Likert scale is functioning as intended, an EFA should indicate that there is only one underlying latent construct being measured. In addition to identifying the number of factors being measured by a Likert scale, an EFA will also identify which items are contributing to the measurement of the latent construct(s) and which are not. In other words, an EFA can reveal how strongly each of the Likert items correlates with the identified factors, which can then suggest whether to remove or add additional items to improve the survey.

### Procedures

To build our standardized tool for assessing supplemental resources in physiology education, our initial step was to identify a prior research effort that had evaluated some digital supplemental resources in medical and allied health education. Browne (18) created and administered a six-item Likert scale to gauge student perception of two, different digital supplemental resources used by undergraduate HAPE students enrolled in various healthcare programs at a 3-yr university. Based on the Association for Medical Education in Europe process for questionnaire developers (35), the survey was created to obtain student feedback about new learning resources that were introduced into a lecture-laboratory learning environment and gauge if the new digital supplemental resources were being used by students and how these new resources functioned for the students.

The Browne survey measured student perception of assisting learning, assisting knowledge retention, and reinforcing academic course content for a collection of objective style physiology quizzes and visually oriented anatomy quizzes. Although the Browne survey functioned as intended by evaluating two different digital supplemental resources in the HAPE setting, the measure could not transfer to another setting or to another digital supplemental resource without modification. One reason was due to slight speech differences, such that the adapted questions might not be understood by a new sample population as intended (36). In this case, modification of the survey was due to cultural differences of the sample population (i.e., different countries) and involved making deliberate vocabulary changes so that the items were stated as clearly as possible for the participants. We adapted the Browne survey for a digital cadaver resource in order to further determine how the survey could be adapted to any digital supplemental resource. We used the same five-point Likert response scale (i.e., strongly agree to strongly disagree) as the original Browne survey for its balance in providing the necessary nuance in participant response without overwhelming the survey participant. Table 1 outlines a summary of the modifications.

**Table 1.** Comparison of Browne survey and the adapted Browne survey

Item Number	Original Measure of Student Opinion about a Supplemental Digital Resource [Browne (18)]	Modified Survey Used in This Study
*1*	The *OPAL quizzes* have helped in my overall understanding of the content in this unit.	The *digital 3 D human body model* (Primal Pictures) has helped me in my overall understanding of anatomy and physiology.
*2*	I used the *OPAL quizzes* as a means of revision before each of my assessments.	I used *Primal Pictures* as a way to study before the tests.
*3*	Feedback from the *OPAL quizzes* helped me to retain the anatomical information.	Feedback from *Primal Pictures* helped me to retain the anatomical information.
*4*	The *OPAL quizzes* helped me to reinforce the information I learnt in the lecture.	*Primal Pictures* helped me to reinforce the information I learned in the lecture.
*5*	The *OPAL quizzes* helped me to reinforce the information I learnt in the anatomy lab.	*Primal Pictures* helped me to reinforce the information I learned in the lab.
*6*	The *OPAL quizzes* were a waste of my time for this unit.	*Primal Pictures* was a waste of my time as a learning tool for this course.

The modified survey was then used in an allied health educational setting, surveying community college students about a different, digital supplemental resource common in HAPE (i.e., digital cadaver). The State University of New York at Buffalo Institutional Review Board determined this research proposal to be exempt. Ninety-nine community college students signed informed consent to be participants in this study. All participants were enrolled in one of four sections of a Human Anatomy and Physiology II lecture-laboratory corequisite course taught by the same instructor. Of this population, 67 participants completed the Likert scale survey, with one participant not completing all items, giving us 66 complete survey responses to analyze.

This population was characteristic of a typical, U.S. community college students in that about half of the students in the course were enrolled in an Allied Health or Nursing degree program, and about half were matriculated in Liberal Arts programs with plans to eventually enter a health care profession. The digital cadaver used in this study was medically accurate and contained several interactive digital human anatomy models. Users could control the digital cadaver with free rotation, zoom in or out, add or remove digital layers or body systems, and toggle labels. Users could also create and customize images using annotation tools and then bookmark or export those images. The digital cadaver was demonstrated by the instructor to all students at the start of the course, and instructions about how to access and use the digital cadaver were housed on the course management system (CMS) for the entire semester. The digital cadaver was also used as a teaching tool during some lectures. For instance, the digital cadaver was used as a visual example during lecture sessions to show anatomical structure and relationships rather than using a traditional two-dimensional still image. These demonstrations increased student exposure to the functionality of the digital cadaver, without promoting use of the digital cadaver directly. Student use of the digital cadaver was strictly voluntary. The digital cadaver was an optional, ungraded tool, to be used at the student’s discretion, on the student’s own time.

The modified survey was administered following the completion of two academic units (i.e., reproductive system and endocrine system) that were taught in a traditional face to face classroom setting. The survey was created and administered using Qualtrics. Distribution of the survey was done by providing an anonymous link to all potential participants by posting the link in the CMS. All students received two points of extra credit on the unit test grade as compensation for completing the survey. Only survey responses from students who gave informed consent were recorded and analyzed. Data was collected over a period of 3 days. The data collected were then analyzed to evaluate the construct validity and reliability of the survey.

Statistical analyses were used to do the following:

1) Examine construct validity of the survey measuring student perception of a supplemental resources.
  a) Examine the correlation among individual Likert items in the survey.
  b) Examine the dimensionality of the survey.
2) Evaluate the internal reliability of the survey.
3) Consider potential improvements to the survey for future iterations.

All of these analyses contribute to determining if the Browne survey could be used as a standardized tool for assessing the value of a digital supplemental resources in medical and allied health education.

## RESULTS

This section focuses on the structure and function of the modified Browne survey. Descriptive statistics were used to examine item response frequencies and how fully the response scale was utilized. Correlation procedures were used to examine individual items and calculate the internal reliability. Exploratory factor analysis (EFA) was used to identify the number of latent constructs being measured by the scale. The number of respondents (*n* = 66) met the minimum necessary for EFA (37). The alpha level for all analyses was 0.05.

### Descriptive Statistics

Table 2 lists the means ± SD and minimum and maximum response values for each of the measured items. Unsurprisingly, the responses show that the participants tend to favor the use of the digital cadaver, revealed by the positively skewed mean for all items. *Item 6* was a negative statement, so it was reverse coded for data analysis purposes. Although this study is not about how students perceive the digital cadaver but rather the functionality of the survey tool, the results in Table 2 indicate that the survey was able to determine an overall positive view of the digital cadaver as a supplemental resource.

**Table 2.** Item means ± SD and minimum and maximum values

Item	Means ± SD	Minimum	Maximum
*1*	1.99 ± 0.81	1	4
*2*	2.42 ± 1.28	1	5
*3*	2.06 ± 0.81	1	4
*4*	2.0 ± 0.94	1	5
*5*	1.97 ± 1.0	1	5
*6* (R)	2.06 ± 1.3	1	5

R, reverse coded.

The frequency of responses is summarized in Table 3 and provides additional detail needed to evaluate of the supplemental resource. In this survey administration, students generally favored the digital cadaver, with over 70% of participants agreeing that the digital cadaver helped their overall understanding of the academic content (Table 3, *item 1*). Responses to *items 4* and *5* reveal that the digital cadaver was equally effective for supplementing both the lecture and laboratory sections of the course. Some responses also indicated that the digital cadaver was not preferred by the entire sample population. For example, the digital cadaver was not considered an effective tool to study for tests by over 20% of the sample population (Table 3, *item 2*). Participants held the strongest opinions about using the digital cadaver as a study tool compared to the other survey items, as evidenced by the lowest percentage of participants being undecided about this issue. There are also responses that could help form future research questions regarding other factors that may influence the effectiveness of the digital cadaver. For example, across *items 1*–*6*, the percentage of participants that remained neutral (i.e., neither agree or disagree) is worthy of further investigation. Perhaps the effectiveness of the digital cadaver is dependent on another factor, e.g., the body system being studied, the type of assessment being used, or even the type of device that the student is using to access the supplemental resource. In addition, over 10% of participants agreed that the digital cadaver was a waste of their time for this course (Table 3, *item 6*). Learning more about the rationale behind this perception could help refine how the instructor recommends use of the digital cadaver in this context. This brief data analysis is intended to demonstrate the type of information that can be ascertained from the survey, as well as the type of follow up questions or future research that the data from the survey can suggest.

**Table 3.** Frequency of responses

Item	*n*	No. of Responses	1Strongly Agree	2Somewhat Agree	3Neither Agree nor Disagree	4Somewhat Disagree	5Strongly Disagree
*1*	99	67	21 (31.3%)	27 (40.3%)	18 (26.9%)	1 (1.5%)	0
*2*	99	67	17 (25.4%)	27 (40.3%)	8 (11.9%)	8 (11.9%)	7 (10.4%)
*3*	99	67	19 (28.4%)	26 (38.8%)	21 (31.3%)	1 (1.5%)	0
*4*	99	66	24 (36.4%)	22 (33.3%)	17 (25.8%)	2 (3%)	1 (1.5%)
*5*	99	67	28 (41.8%)	18 (26.9%)	17 (25.4%)	3 (4.5%)	1 (1.5%)
*6* (R)	99	67	34 (50.7%)	9 (13.4%)	16 (23.9%)	2 (3%)	6 (9%)

R, reverse coded.

Overall, examination of the frequency of responses table allows researchers to see if there is an item that is an outlier in terms of participant responses or lack of responses, which could indicate a problematic item such as an item that was interpreted differently from its intent. Consistency among how the participants answered *items 1*–*6* indicates there is not an item with a higher difficulty level that participants were unable or unwilling to answer that should be considered for removal/revision from the survey.

### Correlation of Items

Since Likert items are designed to measure different aspects of the same latent construct, evidence of statistical correlation using the Pearson correlation coefficient for each bivariate pair verifies the reliability of the survey. Individual items should be positively correlated, but not so strongly that the *r* value suggests multicollinearity, *r* > 0.8 (37), or that the items are identical (i.e., there is no value to repeating the same question). Weak correlations (*r* < 0.3) are also problematic because they suggest that an item does not match with the other items (i.e., off-topic questions distract from what you want to measure) (38, 34). The analysis found that all six modified Likert items were all positively correlated (Table 4). *Items 1* through *5* were appropriately correlated with *r* values ranging between 0.641 and 0.792, suggesting that they were asking about the same latent construct but not repeating any question. The correlation coefficients for *item 6* with all other items (*1*–*5*) fell below an acceptable level of *r* (i.e., 0.3). This suggests that *item* 6 was asking about a construct different than *items 1*–*5*. Another possible explanation for the low correlation coefficient is that *item 6* was misinterpreted by participants (e.g., read too quickly, reading ability) (39).

**Table 4.** Inter-item correlation matrix

	The *digital 3D human body model* (*Primal Pictures*) has helped me in my overall understanding of anatomy and physiology.	I used *Primal Pictures* as a way to study before the tests.	Feedback from *Primal Pictures* helped me to retain the anatomical information.	*Primal Pictures* helped me to reinforce the information I learned in the lecture.	*Primal Pictures* helped me to reinforce the information I learned in the lab.	*Primal Pictures* was a waste of my time as a learning tool for this course.
The *digital 3 D human body model* (*Primal Pictures*) has helped me in my overall understanding of anatomy and physiology.	1.000	0.757	0.731	0.689	0.703	0.137
I used *Primal Pictures* as a way to study before the tests.	0.757	1.000	0.643	0.670	0.697	0.081
Feedback from *Primal Pictures* helped me to retain the anatomical information.	0.731	0.643	1.000	0.742	0.641	0.075
*Primal Pictures* helped me to reinforce the information I learned in the lecture.	0.689	0.670	0.742	1.000	0.792	0.246
*Primal Pictures* helped me to reinforce the information I learned in the lab.	0.703	0.697	0.641	0.792	1.000	0.207
*Primal Pictures* was a waste of my time as a learning tool for this course.	0.137	0.081	0.075	0.246	0.207	1.000

Another way to verify the internal reliability of a Likert scale is through interitem correlation consistency. The greater the interitem consistency, the more likely it is that individual items are measuring the same latent trait (33). The most common, preferred method to estimate reliability for a multiple category response scale is Cronbach’s alpha. The scale showed high internal reliability, Cronbach’s alpha = 0.839. Additional analysis showed that the internal reliability could be improved (Cronbach’s alpha to increase to 0.913 from 0.839) if *item 6* is removed. This finding coincides with the low correlation of *item 6* with the other items.

### Dimensionality

Survey data were analyzed using EFA to see if it was fulfilling its intended design to measure the single construct of student perceived effectiveness of the supplemental resource. Just because it had suitable correlations and alpha levels, the survey could still be measuring multiple constructs (i.e., the items are measuring multiple factors), which would be problematic because a multidimensional survey is analyzed and interpreted differently than a unidimensional survey. The Kaiser-Meyer-Olkin (KMO) measure was used to verify the sampling adequacy, KMO = 0.845. A significant Bartlett’s test of sphericity also confirmed that the data was factorable, *P* < 0.001. Only one factor was detected using Kaiser’s criterion of 1 (40). Therefore, one latent concept (identified as factor 1) underlies the six-item scale and explains over 64% of the variance present in the data (Table 5).

**Table 5.** Total variance explained

	Initial Eigenvalues			Extraction Sums of Squared Loadings		
Factor	Total	%Variance	%Cumulative	Total	%Variance	%Cumulative
*1*	3.867	64.443	64.443	3.568	59.472	59.472
*2*	0.994	16.570	81.013			
*3*	0.388	6.472	87.485			
*4*	0.360	5.999	93.484			
*5*	0.227	3.775	97.259			
*6*	0.164	2.741	100.000			

Extraction method: principal axis factoring.

The latent construct being detected by EFA can be described by the variables with high loading factors. Researchers can also use loading factors to identify items that have low correlation with the extracted factor and therefore do not describe the extracted factor(s). Examination of the factor matrix showed that five of the six items strongly load onto the one extracted factor. This means that five of the six items in the survey are contributing to the measurement of the one identified latent construct. The loading values for items one through five ranged between 0.875 and 0.809, and a loading value >0.722 can be considered significant for a small sample size (41). *Item 6* did not significantly load onto the extracted factor (0.179) and therefore does not significantly describe the latent construct being measured by the other five items in the survey.

The factor matrix identified the five items in the survey that were measuring the effectiveness of the digital supplemental resource in question. HAPE is a subject that is traditionally taught using two modalities: lecture and laboratory. Two of the top three loading items (*items 4* and *5*) measure the degree of reinforcement provided by the supplemental resource for each of these modalities. In addition, the other item in the top three loading factors (*item 1*) describes a reinforcement of overall understanding of course content provided by the supplemental resource. The effectiveness of the supplemental resource can also be described as having the attributes of a study tool used for both test preparation and information retention (*items 2* and *3*). Considering these characteristics, the authors view the measurement being made by the survey as a measure of student perceived effectiveness of the supplemental resource as a learning tool.

## DISCUSSION

The goal of our research was to devise an improved, standardized (i.e., reliable and valid) survey to be used and adapted by physiology researchers and educators to determine student perception of a digital supplemental resource. This knowledge could be used by educators to inform decisions about which supplemental resources are effective for various learning purposes, thereby guiding recommendations to students. Strategic recommendations from educators for supplemental resources could save students time and money since learners may need assistance from educators to gauge which supplemental resources are reliable and effective.

Our analysis does definitively establish that a standardized measure of student perceived value of supplemental resource is both possible and probable. Also, as a result of our analysis, we suggest that our survey can be improved. Individual item analysis and EFA both confirm that *item 6*, a negatively worded statement, was problematic. The correlation between this item and all other items was low (*r* = 0. 246). *Item 6* did not load strongly onto the extracted factor, meaning this item does not contribute to the measurement of the latent construct in a meaningful way. In addition, Cronbach’s alpha is estimated to increase from 0.839 to 0.913 by deleting *item 6* from the survey. This finding is consistent with the fact that combining positively and negatively worded statements within a Likert scale can negatively affect the measure being unidimensional (42).

The aforementioned improvement to the survey was incorporated into a new proposed tool, referred to as the Baptiste-Browne (BB) survey (Table 6). This improved survey can be used as a standardized measurement of student perceived effectiveness of a digital supplemental resource, whether in a learning environment like a physiology lecture-laboratory corequisite course or in an adjacent subject (e.g., biology, microbiology, biomedical sciences, various allied health fields).

**Table 6.** Improved Baptiste-Browne survey

Item	Adaptable Items with *Interchangeable Supplemental Resource in Italics*
*1*	The *supplemental resource* has helped me in my overall understanding of anatomy and physiology.
*2*	I used the *supplemental resource* as a way to study before the test(s).
*3*	Feedback from the *supplemental resource* helped me to retain the anatomical information.
*4*	The *supplemental resource* helped me to reinforce the information I learned in the lecture.
*5*	The *supplemental resource* helped me to reinforce the information I learned in the lab.

Summary of response choices: 5-point ordinal response survey. 1: strongly agree; 2: somewhat agree; 3: neither agree nor disagree; 4: somewhat disagree; 5: strongly disagree.

Two steps are necessary to adapt the BB survey for use. First, replace the term “*supplemental resource*” in each of the five items with the resource of interest (Table 6). For example, if you are looking for student feedback on a new digital resource, or even a resource that is being developed, refer to the resource in a way that will be easily recognizable to the participants completing the survey. Second, update the language in each item if needed to be grammatically correct and to match the vernacular present in the learning environment. Language modifications that change the intent or meaning of an item should be avoided because they could change the established validity of the scale (43). An example in our instrument would be *item 2* (Table 6). If the phrase, “before the test(s)” was removed from the survey, then it would be possible that the question could be interpreted by some students as not referencing the physiology course where the survey was administered. This could then reduce the relevance of answers to the item.

The data obtained from the BB survey can be used by individual instructors to compare the effectiveness of two or more digital supplemental resources. To do a comparison, modify the entire BB survey for each supplemental resource of interest and administer the surveys. Since the BB survey is shown to be unidimensional, each participant’s responses to the five survey items can be summed or averaged, and then the mean score for each supplemental resource can be calculated and compared. This type of analysis can inform instructors about how a newer digital resource compares to a more established resource in a specific course or classroom. It could also assist with decisions about which supplemental resource(s) to recommend or provide within that educational setting. In addition, the BB survey could provide data to compare the effectiveness of an identical form of supplementation for different student populations (e.g., community college students, medical students, allied health students). Individual educators or administrators could use the data to justify funding a particular supplemental resource.

Data obtained from the BB scale could also be utilized to analyze individual items. This type of analysis can identify strengths and weaknesses of a particular supplemental resource (i.e., assisted learning overall, best for retention/study purposes, effectiveness in lecture versus laboratory). To do this, examine the skewness of the means for each item (Table 2). The more positively skewed means (i.e., closer to 1) reflect a greater degree of positive perception for that item while the more negatively skewed means (i.e., closer to 5) can be interpreted as a more critical perception of that item. For example, the survey administration analyzed in this study reveals that participants perceive *item 2* to be least representative of the digital cadaver (Table 2). So even though the participants viewed the digital cadaver positively, they did not necessarily use it to study before a test. This information is useful since it gives student feedback about how the supplemental resource was used, informing the educator whether or not the supplemental resource served the intended purpose. This type of data could inform strategic use of supplementation, which is relevant to instructional designers and educators alike. It could also be useful for authoring future iterations of the digital supplemental resource.

We also note that use of a standardized survey would be superior to educators creating a customized survey tool for each instance of supplemental resource use because, despite the best intentions of the design, without standardization then a survey may be measuring a different construct than intended or multiple unidentified latent constructs.

The BB survey can be adapted and used for applied educational research or for more practical classroom decision making. The BB survey can be used alone or included as part of a longer survey, for example, included with questions about frequency of use. Future steps for survey validation include administering the survey and performing confirmatory factor analysis on the data to establish the survey as a Likert scale. Also, as the survey is utilized, further knowledge about the functionality of each item and the total score can be established. Administration of the survey could also be used to inform the proposed adaptability of the tool.

### Limitations

The findings of this study are limited based on the data being collected from a relatively small, convenience sample. The nonrandom selection of participants can theoretically account for a potential experimental finding (i.e., selection bias), although we do not believe this is the case because demographic data collected on the sample population showed the participants to be representative of community college students nationally. The data analysis performed and the subsequent interpretation of results account for the small, yet adequate, sample size of this study. Regardless, any potential threat based on the sample can be addressed in future studies by either using a large sample size or by using random selection of participants or using both of these methods.

Also, the survey has not been evaluated outside of anatomy and physiology education, higher education, or using traditional nondigital supplemental resources. Further analysis is warranted outside of these parameters. However, we still believe that adoption of the survey for another learning context that utilizes lecture and laboratory course corequisites will lead to useful data because the survey has been successfully utilized for multiple digital resources and has provided accurate data regarding student perception about the effectiveness of digital supplemental resources, which is applicable to any learning context.

Another possible limitation of this study is that the improved BB survey has not yet been implemented at large, only proposed. Although the predicted reliability is established, this tool is meant to be capable of evolving; necessary for use in the rapidly changing world of digital supplementation in medical and allied health education fields. Use of the survey at this stage of development is appropriate since the BB survey does not generate data that have implications for individual participants (i.e., predicting individual student outcomes, influencing placement decisions) (33). If the data collected from the BB survey impacted individual students, then a more rigorous validation process involving analysis of multiple iterations of survey data would be necessary before use.

### Conclusions

The development of new, innovative digital supplemental resources represents a continuing challenge for physiology educators and researchers. From the use of quick response codes (44) to the use of virtual reality (45), it is difficult to know which newer forms of digital supplementation might play a role in student success. This study represents a significant contribution to physiology education by providing a survey that will help instructors decide whether/which digital supplemental resource(s) to recommend to students, a critical but underserved part of student learning. The new proposed survey is short and utilitarian by design, easy to modify and implement for instructors and researchers. Data generated by such a tool are becoming increasing important in an educational environment where new technological tools are created faster than research findings can define their value, or lack thereof, for learning within specific classrooms (46).

## ETHICAL APPROVALS

All research was conducted according to the principles outlined in the Declaration of Helsinki and the Code of Federal Regulations, Title 45: Public Welfare, Part 46: Protection of Human Subjects. The State University of New York at Buffalo Institutional Review Board) determined this research proposal to be exempt. All participants provided informed consent.

## DATA AVAILABILITY

The data that support this study are not publicly available as to protect the privacy of participants but are available from the corresponding author on reasonable request.

## DISCLOSURES

No conflicts of interest, financial or otherwise, are declared by the author(s).

## AUTHOR CONTRIBUTIONS

Y.M.B., S.A., and C.J.B. conceived and designed research; Y.M.B. performed experiments; Y.M.B., S.A., and C.J.B. analyzed data; Y.M.B., S.A., and C.J.B. interpreted results of experiments; Y.M.B. and S.A. drafted manuscript; Y.M.B., S.A., and C.J.B. edited and revised manuscript; Y.M.B., S.A., and C.J.B. approved final version of manuscript.
